# Tissue microarray analyses of the essential DNA repair factors ATM, DNA-PKcs and Ku80 in head and neck squamous cell carcinoma

**DOI:** 10.1186/s13014-024-02541-3

**Published:** 2024-10-30

**Authors:** Henrike Barbara Zech, Clara von Bargen, Agnes Oetting, Nikolaus Möckelmann, Christina Möller-Koop, Melanie Witt, Nina Struve, Cordula Petersen, Christian Betz, Kai Rothkamm, Adrian Münscher, Till Sebastian Clauditz, Thorsten Rieckmann

**Affiliations:** 1 Department of Otorhinolaryngology, University Medical Center Hamburg-Eppendorf, Hamburg, Germany; 2 Institute of Pathology, University Medical Center Hamburg-Eppendorf, Hamburg, Germany; 3 Department of Radiotherapy and Radiation Oncology, University Medical Center Hamburg-Eppendorf, Hamburg, Germany; 4 Department of Otorhinolaryngology, Marienkrankenhaus, Hamburg, Germany; 5 Mildred-Scheel Cancer Career Center HaTriCS4, University Medical Center Hamburg-Eppendorf, Hamburg, Germany

**Keywords:** Ku80, DNA-PKcs, ATM, HNSCC, Tissue microarray, Prognostic marker, DNA repair, Non-homologous endjoining

## Abstract

**Background:**

Head and neck squamous cell carcinoma (HNSCC) negative for Human Papillomavirus (HPV) has remained a difficult to treat entity, whereas tumors positive for HPV are characterized by radiosensitivity and favorable patient outcome. On the cellular level, radiosensitivity is largely governed by the tumor cells` ability to repair radiation-induced DNA double-strand breaks (DSBs), but no biomarker is established that could guide clinical decision making. Therefore, we tested the impact of the expression levels of ATM, the central kinase of the DNA damage response as well as DNA-PKcs and Ku80, two major factors in the main DSB repair pathway non-homologous end joining (NHEJ).

**Methods:**

A tissue microarray of a single center HNSCC cohort was stained for ATM, DNA-PKcs and Ku80 and the expression scored based on staining intensity and the percentages of tumor cells stained. Scores were correlated with clinicopathological parameters and survival.

**Results:**

Samples from 427 HNSCC patients yielded interpretable stainings and were scored following an established algorithm. The majority of tumors showed strong expression of both NHEJ factors, whereas the expression of ATM varied more. The expression scores of ATM and DNA-PKcs were not associated with patient survival. For HPV-negative HNSCC, the minority of tumors without strong Ku80 expression trended towards superior survival when treatment included radiotherapy. Focusing stronger on staining intensity to define the subgroup with lowest and therefore potentially insufficient expression levels in the HPV-negative subgroup, we observed significantly better overall survival for patients treated with radiotherapy but not with surgery alone.

**Conclusions:**

Our data suggest that HPV-negative HNSCC with particularly low Ku80 expression represent a highly radiosensitive subpopulation. Confirmation in independent cohorts is required.

**Supplementary Information:**

The online version contains supplementary material available at 10.1186/s13014-024-02541-3.

## Introduction

Radio(chemo)therapy, either after surgery or in the primary setting, is a mainstay of curative treatment for locally advanced head and neck squamous cell carcinoma (HNSCC). Apart from Human Papillomavirus (HPV) and p16 expression, which mark a biologically distinct, radiosensitive subgroup of oropharyngeal tumors (OPSCC) [1, 2], no molecular markers have been established that could guide therapeutic decision making. For HPV-positive OPSCC, various strategies for treatment de-intensification have been and are further being tested. For HPV-negative HNSCC, cure rates are still unsatisfactory and treatment intensification would be desirable but current multimodal regimes are already operating at the maximum tolerable level. Therefore, biomarkers that could prospectively identify patients at high or low risk of treatment failure in both subgroups are highly desirable.

Tumor cell killing after radiotherapy is primarily caused through the induction of DNA lesions, of which DNA double-strand breaks (DSBs) are the most deleterious. These lesions activate a specialized signaling network, called the DNA damage response (DDR), which recognizes the breaks, recruits and activates DSB repair factors, and provides additional repair time by attenuating cell cycle progression. A central component of the DDR is the PI3K-like kinase ataxia telangiectasia mutated (ATM). Germline loss-of-function mutations of ATM are the cause for ataxia telangiectasia syndrome. Apart from the name-providing features, the disease is also characterized by early tumor development and severe radiosensitivity caused by impaired DSB repair as assessed by enhanced levels of unrepaired DSBs persisting for prolonged times after irradiation [3–5]. Copy number losses of the distal arm of chromosome 11, which includes the ATM gene locus, are frequently observed in HNSCC in general and even more so in the more radiosensitive HPV-positive OPSCC [6]. Notwithstanding a potentially reduced expression of ATM, Lim et al. have not observed an association with patient outcome after chemoradiation treatment [7] and others even reported inferior outcome for patients and enhanced radioresistance in HNSCC cell lines with loss of distal 11q despite the controversial finding of an attenuated DDR [8, 9]. The actual repair processes downstream of the DDR are mainly performed through the DSB repair pathway of classical non-homologous endjoining (NHEJ), the dominant DSB repair pathway in all cell cycle phases [10, 11]. Therefore, efficient NHEJ is also prerequisite for radioresistance, and the inhibition of expression or function of essential factors, such as Ku70/80, DNA-PKcs or DNA ligase IV results in very pronounced cellular radiosensitivity [12–14].

In the clinical setting, the association between the expression levels of DDR and NHEJ factors and patient outcome after radiotherapy has been performed in a variety of cancer types, such as cervix or prostate cancer, but also others, with some promising but also contradictory results over the last two decades [15–22]. Studies on the association of ATM and the core NHEJ factors DNA-PKcs and Ku70/80 with patient outcome in HNSCC, have so far also reported conflicting results. Despite their critical roles in DSB repair, Friesland et al. have reported that high expression of DNA-PKcs/Ku80 in combination with p53 negativity in tonsillar carcinoma was associated with better survival of patients treated with radiotherapy, suggesting radiosensitivity [23]. Similarly, Pavon et al., showed that high KU70 mRNA levels and a higher fraction of tumor cells stained positive for Ku80 and especially Ku70 were associated with improved responses towards induction chemotherapy (ICT) and improved local recurrence-free survival (LRFS) after subsequent radiotherapy. High levels of DNA-PKcs were also associated with ICT responses but not LRFS in that study [24]. In contrast, Lee et al. observed significantly higher 5-year locoregional control rates in patients with low Ku70 expression in nasopharyngeal cancer treated with R(C)T, whereas expression of DNA-PKcs did not yield significant differences [25]. Similarly, Joshi et al. recently reported of higher survival rates in oral SCC patients whose tumors demonstrated lower Ku80 mRNA levels but significance was lost in a multivariate analysis and only remained for the protein level of XRCC4, another NHEJ factor [26]. The most comprehensive analysis of DNA repair factors in HNSCC in a clinical setting was performed by Moeller et al. in 2011 [27]. The authors compared the expression levels of 18 DSB repair factors and 19 oncologically relevant proteins not primarily related to DSB repair in a cohort of 89 chemoradiation-treated HNSCC patients and also took the tumors` HPV-status into account. In this thorough analysis, the NHEJ repair factor Ku80 was identified as the by far most relevant predictor of patient survival, whereas DNA-PKcs and ATM expression had little effect. High Ku80 expression was associated with poor outcome in patients with HPV-negative tumors suggesting radioresistence, and this result was recapitulated in a validation cohort of 34 independent HPV-negative patients. However, no related publications have either confirmed the data or shown contradictory results and the Ku80 expression level has never entered the clinic as a prognostic or predictive biomarker. Against this background we analyzed the expression of the central DDR and DSB repair proteins ATM, Ku80 and DNA-PKcs in a retrospective cohort of 427 HNSCC patients.

## Materials and methods

### Patient material

In this study we retrospectively analyzed survival and the clinicopathological data from patients, who had been diagnosed with squamous cell carcinoma of the head and neck and treated with curative intent at the University Medical Center Hamburg-Eppendorf between 1992 and 2013 with a vast majority of 95% diagnosed between 2000 and 2013. Tissues were obtained from primary tumors during surgical resection or diagnostic pretreatment biopsies. Patient consent was waived because the use of archived remnants of diagnostic tissues and their analysis for research purposes as well as patient data analysis have been approved by local laws (HmbKHG,§ 12,1) and by the local ethics committee (Ethics commission Hamburg, WF-049/09). The whole study has been carried out in compliance with the Helsinki Declaration.

### Tissue microarray construction

Tissue samples were fixed in buffered 4% formalin, embedded in paraffin, and used for tissue microarray (TMA) construction as previously described [28, 29]. Haematoxylin-eosin stained sections were made from each selected primary tumor block to identify representative tumor regions. One tissue cylinder (0.6 mm in diameter) was punched from each tumor using a homemade semi-automated tissue arrayer. For immunohistochemical staining, three-micrometer TMA sections were prepared using the Paraffin Sectioning Aid System (Instrumentics, Hackensack, NJ).

### Immunohistochemistry

For immunohistochemistry (IHC) analyses freshly cut 3 μm thick TMA sections were analyzed on the same day in a single experiment. ATM, DNA-PKcs, Ku80 and p16 were stained using specific antibodies (rabbit anti-ATM (Abcam, #ab32420, clone Y170, 1:450); mouse anti-DNA-PKcs (Sigma, #SAB1404245, clone 2A8, 1:1350); mouse anti-Ku80 (Abcam, #ab119935, clone 5C5, 1:4050); mouse anti-p16 (BD Biosciences, clone G175-405, 1/3600) after peroxidase blocking with H_2_O_2_ (DAKO S2023) for 10 min. High-temperature pretreatment of slides was done in an autoclave with citrate buffer, pH 7.8 for 5 min. The Envision system (DAKO5007) was used to visualize the immunostaining.

The staining was categorized using a well-established scoring system based on staining intensity (0, 1, 2, 3 - referring to absent, low, intermediate or high intensity) and the fraction of tumor cells stained [30, 31]. The final IHC score (negative, weak, moderate, strong) is built from these parameters as follows: negative scores had a staining intensity of 0; weak scores had a staining intensity of 1 in ≤ 70% of tumor cells or a staining intensity of 2+ in ≤ 30% of tumor cells; moderate scores had a staining intensity of 1+ in > 70% of tumor cells, a staining intensity of 2+ in > 30% and ≤ 70% of tumor cells and a staining intensity of 3 in ≤ 30% of tumor cells; strong scores had a staining intensity of 2+ in > 70% of tumor cells or a staining intensity of 3 in > 30% of tumor cells. In an alternative score (low or intermediate/high) with stronger focus on staining intensity, low scores had a staining intensity of 2 or 3 in ≤ 30% of tumor cells, and intermediate/high scores had a staining intensity of 2 or 3 in > 30% of tumor cells.

p16 status was scored as positive when ≥ 70% of tumor cells demonstrated moderate or strong staining intensity. For sample pictures, the TMA slides were scanned on a digital whole slide scanner (Aperio AT2, Leica) at 40x magnification.

### Data analyses

R (version 3.6.3) and Bioconductor environment [32] were used for data processing, analysis and evaluation. Survival analyses were performed according to the Kaplan-Meier method and the Log-rank test. Multivariable analyses were performed fitting a Cox proportional hazards regression model (R-packages: survival and survminer) [33, 34]. Potential associations between variables were tested using the Pearson correlation coefficient (R-packages: reshape and corrplot) [35, 36]. All statistical analyses are to be considered exploratory. The reported p-values are two-sided and used as descriptive measures only. The depiction of TMA scores was performed using GraphPad Prism 6.

## Results

We assessed the protein expression level of the critical DSB repair factors ATM, DNA-PKcs and Ku80 in a tissue microarray (TMA) from patients treated with curative intent at the University Medical Center Hamburg-Eppendorf. Interpretable stainings were obtained in 427 individual samples. p16 status as an indicator for either HPV-induced or HPV-independent tumorigenesis was available for 172 of 202 OPSCC samples. 340 patients (79.7%) were treated primarily by surgery. Of these 142 received surgical treatment alone (33.3%) and 198 received adjuvant radiotherapy or radiochemotherapy (RT/RCT) (46.4%). Sixty-nine patients were primarily treated with RT/RCT (16.1%). Patient characteristics are shown in Table 1.

**Table 1:** Clinicopathological characteristics. T- and N-classification were performed according to the 7th edition of the Union for International Cancer Control (UICC). Classification represents pathological staging for resected tumors and clinical staging for tumors treated with definitive RT/RCT. p16 status was not available (n.a.) for 30 OPSCC specimen, because of a lack of tumor tissue in the p16-stained tissue microarray section or because of indistinct scoring (intermediate or high staining intensity in >40 but <70% of tumor cells)

Patient characteristics	
**Interpretable staining**, number (%)	
ATM and/or DNAPKcs and/or KU80	427 (100)
ATM	396 (93)
DNA-PKcs	381 (89)
Ku80	343 (80)
**Age**, median (range)	61 (32–85)
**Sex**, number (%)	
male	334 (78.2)
female	93 (21.8))
**Location**, number (%)	
Oropharynx	202 (47.3)
p16+ (% of OPSCC)	70 (34.7)
p16- (% of OPSCC)	102 (50.5)
p16 n.a. (% of OPSCC)	30 (14.9)
Larynx	136 (31.9)
Hypopharynx	57 (13.3)
Oral cavity	26 (6.1)
Nasopharynx	6 (1.4)
**T classification**, number (%)	
T1	105 (24.6)
T2	118 (27.6)
T3	100 (23.4)
T4	102 (23.9)
n.a.	2 (0.5)
**N classification**, number (%)	
N0	159 (43.9)
N1	53 (14.6)
N2	131 (36.2)
N3	19 (5.3)
**Therapy**, number (%)	
surgery	142 (33.3)
surgery + (chemo)radiation	198 (46.4)
chemoradiation	57 (13.3)
radiotherapy	12 (2.8)
other	12 (2.8)
n.a.	6 (1.7)

### Expression of DSB repair factors

**Fig. 1:**
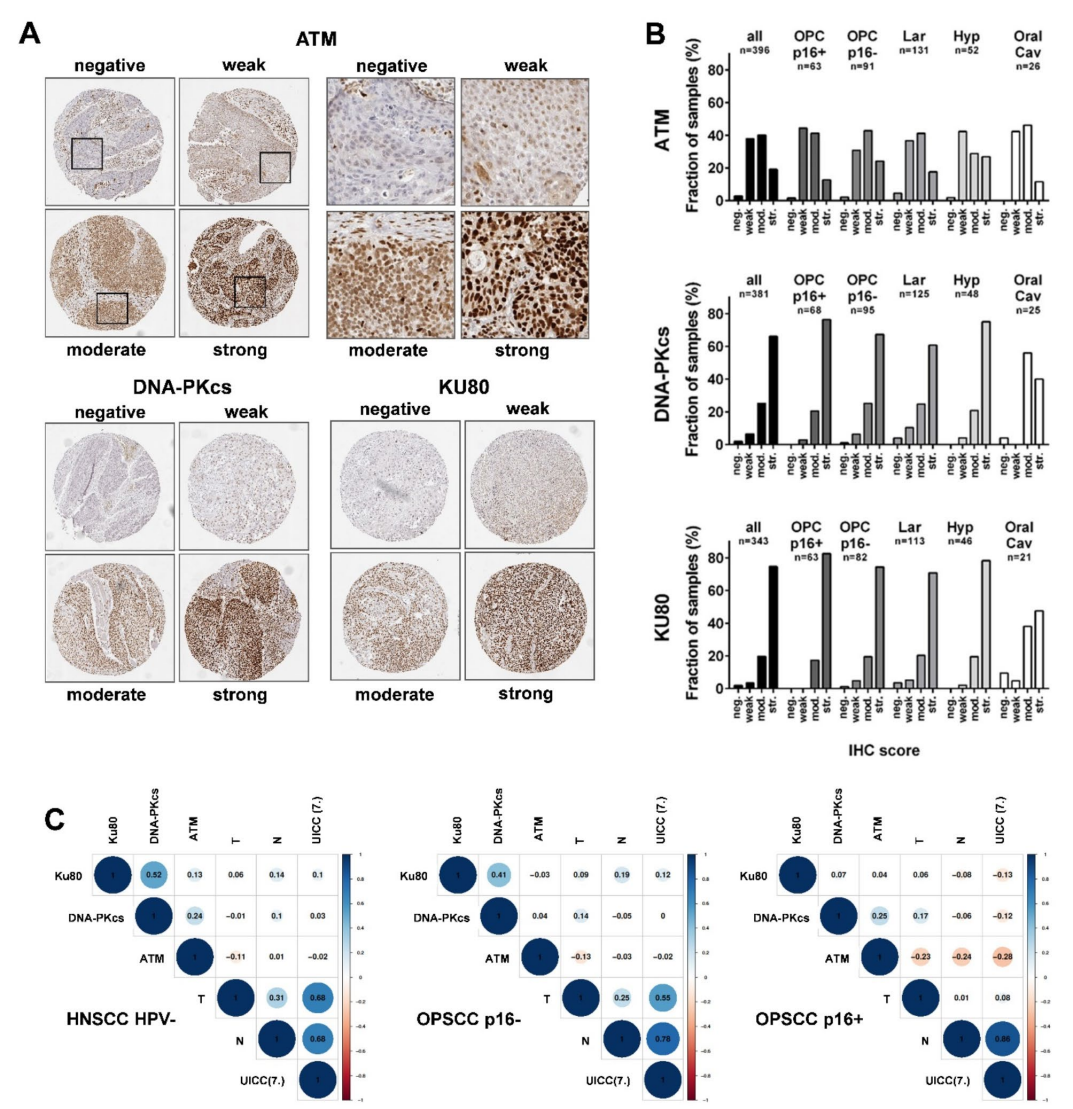
Immunohistochemical staining. (**A**) Representative examples of TMA score categories. Magnified areas demonstrate nuclear expression, which was observed for all three proteins in virtually all samples. (**B**) Distribution of TMA scores in the whole group and in specific subtypes. (**C**) Correlation analyses of TMA scores and T & N stages. UICC-stage (7th edition) served as an internal control and, as expected, was vastly governed by N stage in HPV-positive OPSCC while correlating with both T & N stage in HPV-negative tumors

In a semiquantitative analysis, the immunohistochemical stainings of ATM, DNA-PKcs and Ku80 were scored as either *negative*, *weak*, *moderate* or *strong* depending on the staining intensity and the respective portion of the tumor cells stained, following a well-established algorithm [30, 31] **(**Fig. 1A**)**. Expression of all three DNA repair factors was vastly restricted to the nucleus in virtually all cases. For the NHEJ proteins DNA-PKcs and Ku80, we observed a similar distribution of protein expression across the HNSCC subsites with a clear majority of samples demonstrating *strong* expression (intermediate staining intensity [2] in >70% or high intensity [3] in >30% of tumor cells), except for the comparably small cohort of oral cavity tumors, in which moderate staining was similarly frequent. ATM staining was clearly less intense than DNA-PKcs and Ku80 staining with *weak* and *moderate* representing the most frequent categories **(**Fig. 1B**)**. For correlation and survival analyses, due to the different etiology of HPV-driven and unrelated tumors, we built a pooled cohort containing p16-negative OPSCC and all non-oropharyngeal cases (except for the few nasopharyngeal samples), in which HPV-driven tumors will represent only a small minority below 5%, when the HPV-status is carefully assessed through detection of HPV E6/E7 mRNA in addition to p16 or HPV DNA [37–42]. Correlation analyses of the staining scores and T + N stage as important clinicopathological characteristics for patient outcome demonstrated a positive association of the NHEJ factors Ku80 and DNA-PKcs in HPV-negative HNSCC and p16-negative OPSCC. No association was found for p16-positive OPSCC, in which samples demonstrating *negative* and *weak* expression of Ku80 were completely missing **(**Fig. 1C**)**.

### Impact of DSB repair factors on patient survival

**Fig. 2:**
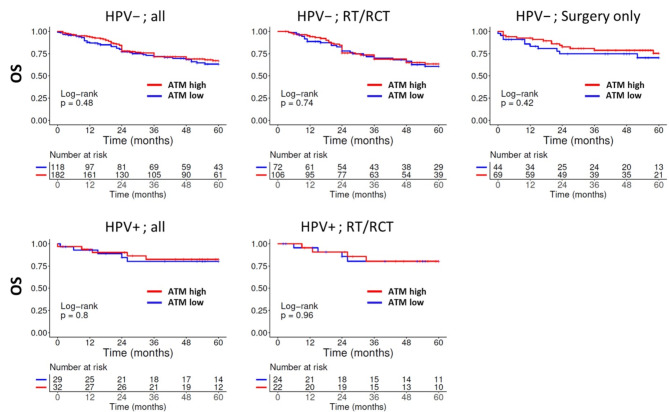
Overall survival in dependence of ATM expression and treatment. ATM-expression was categorized by staining scores as *low* (*absent & weak*) or *high* (*moderate & strong*). The low numbers of p16-positive OPSCC treated solely by surgery prevented a meaningful analysis

*ATM*: To assess the impact of the expression level of the central DDR kinase ATM on patient survival we dichotomized our cohort into patients with tumors showing low (*absent* & *weak* staining score) vs. high (*moderate* & *strong* staining score) expression. We did not observe any influence on overall or recurrence free survival (OS, RFS), neither in the pooled cohort of patients with HPV-negative tumors nor in those with p16-positive OPSCC. Stratification by therapeutic treatment also did not reveal any impact of the ATM expression level on patients treated with or without radio(chemo)therapy in any form (primary and adjuvant) or by surgery only (Fig. 2, Supplementary Fig. 1). Alternative dichotomization strategies as well as analyses of specific anatomical subgroups or only of patients primarily treated by RT/RCT also did not demonstrate any prognostic impact of ATM expression levels (not shown).

*DNA-PKcs and Ku80*: Due to the high expression levels of the NHEJ factors DNA-PKcs and Ku80 with *strong* staining scores being the by far most frequent category (see Fig. 1B), we assorted patients into those demonstrating *strong* vs. all other stainings for survival analyses.

**Fig. 3:**
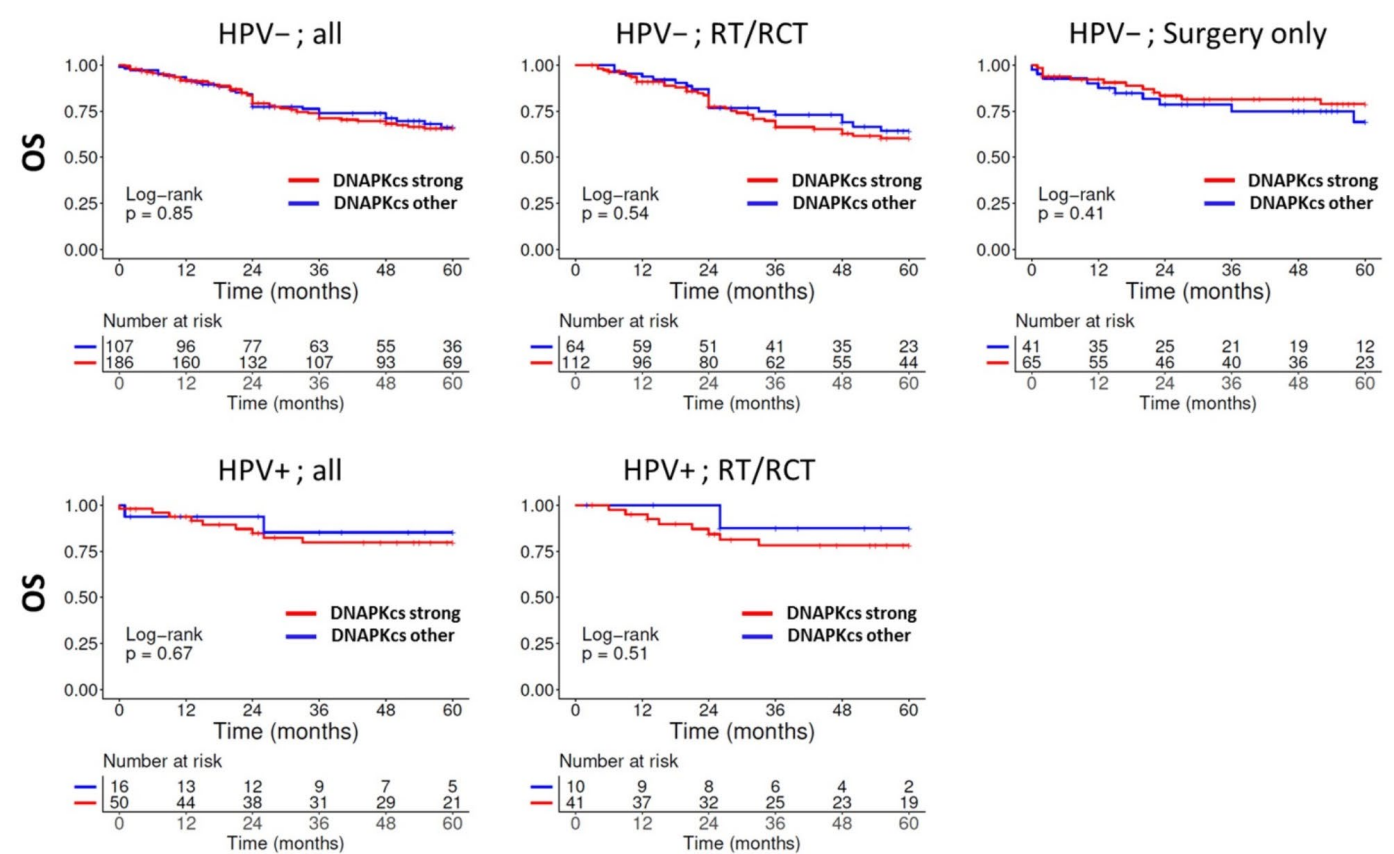
Overall survival in dependence of DNA-PKcs expression and treatment. Expression was categorized by staining scores as *strong* or *other*. The low numbers of p16-positive OPSCC treated solely by surgery prevented a meaningful analysis

Similar to the analysis of ATM, we did not observe an impact of DNA-PKcs expression levels on patient OS or RFS, neither in HPV-negative HNSCC, p16-positive OPSCC and independent of treatment (Fig. 3 and Supplementary Fig. 2) and tumor sublocalisation (not shown).

**Fig. 4:**
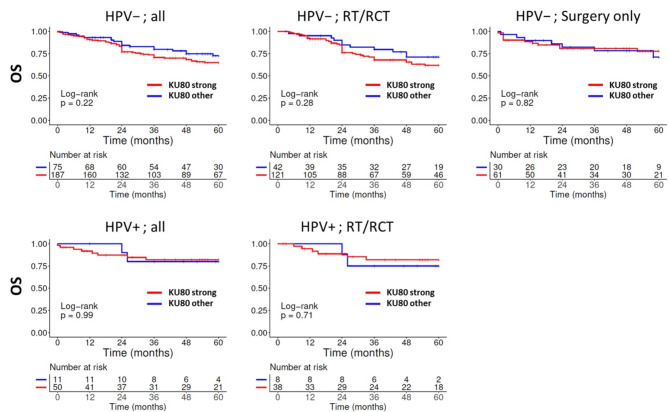
Overall survival in dependence of Ku80 expression and treatment. Expression was categorized by staining scores as *strong* or *other*. The low numbers of p16-positive OPSCC treated solely by surgery prevented a meaningful analysis

For Ku80, we observed a trend towards inferior overall and recurrence free survival for tumors with strong expression in HPV-negative HNSCC. This effect was driven by tumors receiving some form of radiotherapy, while there was no such trend in those treated solely by surgery. However, in contrast to Moeller et al. [27], who used a similar scoring system, the effects of different Ku80 staining scores in our analyses were very modest and did not reach statistical significance (Fig. 4 and Supplementary Fig. 3).

**Fig. 5:**
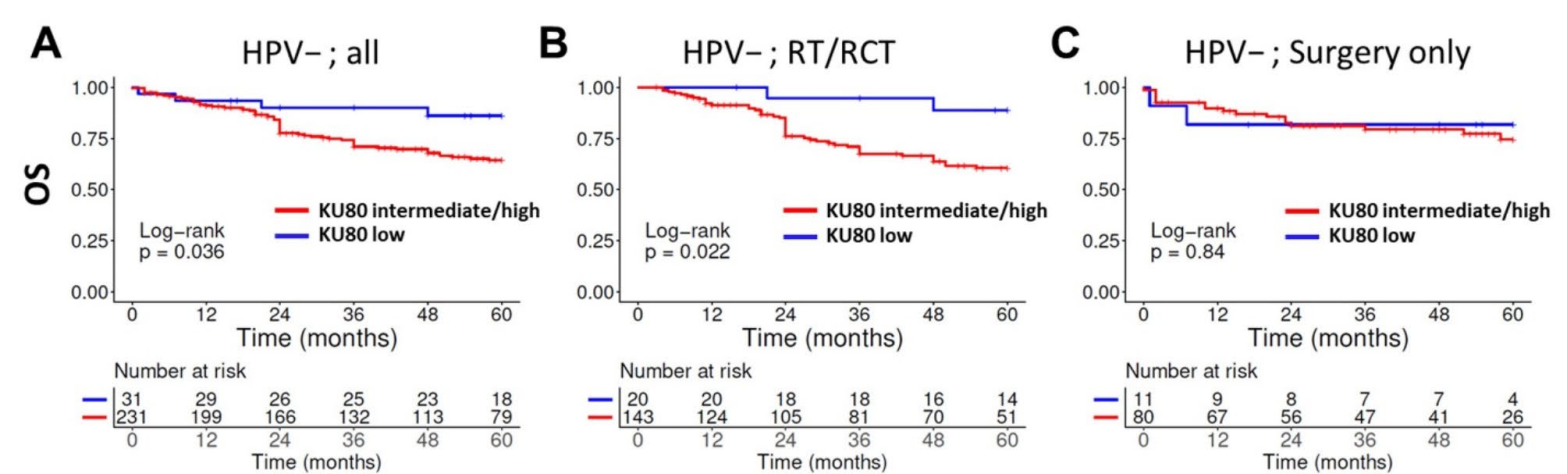
Overall survival in dependence of Ku80 staining intensity and treatment. Expression was categorized by staining intensities as either *low* or *intermediate/high* with a threshold of > 30% of tumor cells with at least intermediate staining intensity (2 or 3) necessary to be assorted to the higher category

DNA-PKcs and Ku80 usually showed quite homogeneous staining patterns, so that tumor samples showing low staining intensities of 1 were in most cases classified as *moderate* in the established scoring system due to the high percentage of tumor cells stained. As a result, tumors scored as *weak* are extremely rare in our analysis (see Fig. 1B). We therefore performed an alternative analysis focussing stronger on staining intensity and less on the fraction of cells stained with the hypothesis that low cellular expression levels of NHEJ factors may confer cellular radiosensitivity because of a less effective main DSB repair pathway. To this end, we defined *low* expressing tumors as those mostly demonstrating staining intensities of 0 and 1 using a threshold of > 30% of cells with higher staining intensities of 2 and 3 necessary for the spot to be scored as *intermediate/high* expression. For DNA-PKcs we did not observe an obvious impact of *low* expression levels (not shown). In contrast, the comparably small subgroup of HPV-negative HNSCC demonstrating *low* Ku80 expression and especially those patients whose treatment included radiotherapy demonstrated significantly superior OS (*p* = 0.036 and *p* = 0.022, respectively) and corresponding trends for RFS (Fig. 5, Supplementary Fig. 4, top). This difference mostly depended on patients treated with primary surgery and adjuvant RT/RCT (*p* = 0.023) (Supplementary Fig. 4, bottom), whereas the small number of only 4 tumors with *low* staining intensity primarily treated by RT/RCT prevented a meaningful analysis. No difference in dependence of Ku80 expression was detected in patients treated by surgery alone.

In a multivariable analysis of patients with HPV-negative tumors treated by radiotherapy in any form, that included Ku80 staining intensity (0,1 vs. 2,3), T- and N-stage, sex and age, Ku80 remained a significant, independent prognostic factor for OS. Significance was further reached for age but slightly missed for T- and N-stage in our cohort (Table 2).

**Table 2:** Multivariable analysis. Asterisks indicate significant associations of variables and OS (Cox proportional hazards regression, * indicates *p* < 0.05). The table includes all tested variables of the respective analyses

Variables	Overall survival		
	HR	95% CI	*p*-value
Ku80 (low)	0.2	0.04809 - 0.8319	* 0.0269
T-stage	1.278	0.96308 - 1.7007	0.0890
N-stage	1.325	0.98649 - 1.7790	0.0615
age	1.42	1.04889 - 1.9224	* 0.0233
sex (m, f)	0.735	0.38384 - 1.4064	0.3521

## Discussion

Our retrospective TMA analysis of a cohort of curatively treated HNSCC patients strongly suggests that the expression level of the central DDR kinase ATM and the NHEJ kinase DNA-PKcs do not possess prognostic value in HNSCC irrespective of HPV-status and involvement of radiotherapy, in line with previous reports [7, 24, 27]. This does not imply that the activation levels of these kinases or of their biological pathways are not important factors in the modulation of radioresponsiveness. This has, for example, been suggested for impaired activation of ATM itself or impaired activity of the ATM-orchestrated DDR for the enhanced radiation sensitivity of HPV-positive HNSCC [43, 44] or for inactivating ATM mutations for some exceptional tumor responses to radiotherapy [45] and may not be assessable by immunohistochemical protein quantification.

Regarding Ku80, our data clearly call into question some older publications, which describe this protein as a positive prognostic factor in HNSCC [23, 24]. For the HPV-negative cohort, our data also do not fully support the finding of Moeller et al. which described strong Ku80 expression as a dominating negative prognostic factor using a highly similar scoring algorithm. In that publication about half of all tumors were scored as strongly expressing, which conferred a profoundly unfavorable prognosis, while patients with low or intermediate expression showed equally high survival rates [27]. Apart from using a different antibody, which may well account for some differences in staining intensities between the two studies, another potentially relevant difference between both cohorts is that in Moeller et al. all 89 patients were treated with primary chemoradiation, whereas the majority of the 267 patients treated with some form of radiotherapy in our study received RT/RCT in the adjuvant setting, which reflects common treatment approaches in the US and in Germany.

While not confirming the Moeller results in detail, our data also show profound differences in patient survival in dependence of Ku80 expression when using an alternative scoring system to identify patients with particularly low expression levels, namely those showing no or only faint Ku80 staining intensity in the majority (≥ 70%) of tumor cells. For patients with HPV-negative HNSCC this small group demonstrated significantly favorable OS and a trend towards improved PFS when treatment included radiotherapy but not after treatment by surgery only. From the biologic point of view, low Ku80 expression is a rational candidate for a marker conferring cellular radiosensitivity and hence superior patient survival after radiotherapy. The Ku70/80 heterodimer is usually highly abundant in mammalian nuclei and binds to DSBs within seconds after their induction [46, 47]. It mediates positional stability of the DSB ends preventing end separation [48], which would make the ends far more susceptible to repair failure or misrepair, both of which can easily give rise to lethal chromosomal aberrations. Furthermore, it helps to protect the DSB ends from DNA end resection, and insufficient protection and subsequent inappropriate end resection may foster a dependency of such cells on the error prone backup repair pathways alternative endjoining (alt-EJ) and single-strand annealing (SSA), as already demonstrated for Ku-deficient cells in vitro [49, 50]. This may be especially crucial for DSBs in G1-phase cells or during S-phase in regions where sister chromatid synthesis has not been completed, both conditions not permissive for faithful repair by homologous recombination (HR). In this case it would be reasonable that low Ku80 expression may also represent a predictive marker for the response towards a combination of radiotherapy and PARP-inhibition, since alt-EJ is PARP-dependent and its inhibition would deplete the cells of a backup repair pathway especially required in these cells. Mechanistically, a recent publication has described radiosensitivity in nasopharyngeal carcinoma mediated by the deubiquitinase USP44 via stabilization of the E3 ubiquitin ligase TRIM25 and subsequent TRIM25-mediated Ku80 degradation. Hypermethylation of the USP44 promoter in nasopharyngeal cancer disrupts this axis, leading to higher Ku80 levels and radioresistance. Consistently, low USP44 levels were significantly associated with profoundly inferior survival in a large cohort of 376 nasopharyngeal cancer patients all treated by RCT [51]. While that study demonstrated compelling evidence for an interrelation of USP44 and Ku80 expression from in vitro and in vivo models, an association of the respective protein levels in clinical cohorts of nasopharyngeal tumors as well as a validation of the mechanism in other HNSCC subgroups still remains to be shown. Given its central role in DNA repair, the Ku70/80 dimer is also being considered as a potential therapeutic target for radio- and chemosensitisation. For example, a recent publication presented the small molecule UMI-77 as a potent Ku inhibitor in a preclinical screening [52]. However, since normal tissue cells use the same DDR and DNA repair factors and mechanisms, it remains to be shown to what extent the inhibition of such integral components as ATM, DNA-PKcs or Ku70/Ku80 will be able to induce radiosensitisation in a sufficiently tumour-specific manner.

Our study has several limitations. The data represent a retrospective cohort analysis and single spot TMA analyses do not cover intratumor heterogeneity. The tumor specimens were collected over a long time frame (95% of tumors treated between 2000 and 2013) with evolving techniques in both surgery and RT. However, since tumor samples with low Ku80 expression were distributed over the entire collection period and the prognosis of HPV-negative HNSCC has hardly changed over this period, we do not expect a relevant bias in survival. Our cohort also lacks HPV status so we rely on p16 as a marker of HPV-induced tumorigenesis in OPSCC. However, in this subsite p16 is a well-established surrogate marker [53] and thorough mRNA-based analyses of the HPV status clearly demonstrated that in non-oropharyngeal subsites, active HPV infections are very rare [37–42]. We therefore think that although single HPV-positive tumors may be included in our pooled HPV-negative cohort, just as single HPV-negative tumors in the group of p16-positive OPSCC, they are most unlikely to substantially confound the results. Finally, while our data represent the analysis of a comparatively large HNSCC cohort, various subgroups consist of a rather limited number of patients, including the most relevant one with HPV-negative tumors showing particularly low Ku80 expression and whose treatment included radiotherapy (*n* = 20). Therefore, our finding of an especially favorable OS in this group has to be considered as hypothesis-generating, and confirmation in further retrospective or, ideally, prospective cohorts is necessary. Further research on the potential of low Ku80 expression as a valid prognostic marker for HNSCC treated with radiotherapy and, if confirmed, on the potential as a predictive marker for PARP-inhibition concomitantly applied with radiotherapy in the frame of personalized medicine concepts in oncology is recommended.

## Conclusion

HPV-negative HNSCC with particularly low Ku80 expression are likely to represent a highly radiosensitive subpopulation. This hypothesis should be confirmed in independent cohorts.

## Electronic supplementary material

Below is the link to the electronic supplementary material.

Supplementary Material 1

## Data Availability

No datasets were generated or analysed during the current study.

## References

[1] Ang KK, Harris J, Wheeler R, Weber R, Rosenthal DI, Nguyen-Tan PF, et al. Human papillomavirus and survival of patients with oropharyngeal cancer. N Engl J Med. 2010;363(1):24–35. 20530316 10.1056/NEJMoa0912217 PMC2943767

[2] Lassen P, Eriksen JG, Hamilton-Dutoit S, Tramm T, Alsner J, Overgaard J. Effect of HPV-associated p16INK4A expression on response to radiotherapy and survival in squamous cell carcinoma of the head and neck. J Clin Oncol. 2009;27(12):1992–8. 19289615 10.1200/JCO.2008.20.2853

[3] Blackford AN, Jackson SP, ATM, ATR DNA-PK. The trinity at the heart of the DNA damage response. Mol Cell. 2017;66(6):801–17. 28622525 10.1016/j.molcel.2017.05.015

[4] Kuhne M, Riballo E, Rief N, Rothkamm K, Jeggo PA, Lobrich M. A double-strand break repair defect in ATM-deficient cells contributes to radiosensitivity. Cancer Res. 2004;64(2):500–8. 14744762 10.1158/0008-5472.can-03-2384

[5] Rothblum-Oviatt C, Wright J, Lefton-Greif MA, McGrath-Morrow SA, Crawford TO, Lederman HM. Ataxia telangiectasia: a review. Orphanet J Rare Dis. 2016;11(1):159. 27884168 10.1186/s13023-016-0543-7 PMC5123280

[6] Hayes DN, Van Waes C, Seiwert TY. Genetic Landscape of Human Papillomavirus-Associated Head and Neck Cancer and comparison to Tobacco-related tumors. J Clin Oncol. 2015;33(29):3227–34. 26351353 10.1200/JCO.2015.62.1086 PMC4586167

[7] Lim AM, Young RJ, Collins M, Fox SB, McArthur GA, Corry J, et al. Correlation of Ataxia-Telangiectasia-Mutated (ATM) gene loss with outcome in head and neck squamous cell carcinoma. Oral Oncol. 2012;48(8):698–702. 22410096 10.1016/j.oraloncology.2012.02.014

[8] Parikh RA, White JS, Huang X, Schoppy DW, Baysal BE, Baskaran R, et al. Loss of distal 11q is associated with DNA repair deficiency and reduced sensitivity to ionizing radiation in head and neck squamous cell carcinoma. Genes Chromosomes Cancer. 2007;46(8):761–75. 17492757 10.1002/gcc.20462

[9] Sankunny M, Parikh RA, Lewis DW, Gooding WE, Saunders WS, Gollin SM. Targeted inhibition of ATR or CHEK1 reverses radioresistance in oral squamous cell carcinoma cells with distal chromosome arm 11q loss. Genes Chromosomes Cancer. 2014;53(2):129–43. 24327542 10.1002/gcc.22125 PMC4216593

[10] Chang HHY, Pannunzio NR, Adachi N, Lieber MR. Non-homologous DNA end joining and alternative pathways to double-strand break repair. Nat Rev Mol Cell Biol. 2017;18(8):495–506. 28512351 10.1038/nrm.2017.48 PMC7062608

[11] Kakarougkas A, Jeggo PA. DNA DSB repair pathway choice: an orchestrated handover mechanism. Br J Radiol. 2014;87(1035):20130685. 24363387 10.1259/bjr.20130685 PMC4064598

[12] Allalunis-Turner MJ, Zia PK, Barron GM, Mirzayans R, Day RS. 3rd. Radiation-induced DNA damage and repair in cells of a radiosensitive human malignant glioma cell line. Radiat Res. 1995;144(3):288–93. 7494872

[13] Marangoni E, Foray N, O’Driscoll M, Douc-Rasy S, Bernier J, Bourhis J, et al. A Ku80 fragment with dominant negative activity imparts a radiosensitive phenotype to CHO-K1 cells. Nucleic Acids Res. 2000;28(23):4778–82. 11095690 10.1093/nar/28.23.4778 PMC115156

[14] Singleton BK, Priestley A, Steingrimsdottir H, Gell D, Blunt T, Jackson SP, et al. Molecular and biochemical characterization of xrs mutants defective in Ku80. Mol Cell Biol. 1997;17(3):1264–73. 9032253 10.1128/mcb.17.3.1264 PMC231851

[15] Bouchaert P, Guerif S, Debiais C, Irani J, Fromont G. DNA-PKcs expression predicts response to radiotherapy in prostate cancer. Int J Radiat Oncol Biol Phys. 2012;84(5):1179–85. 22494583 10.1016/j.ijrobp.2012.02.014

[16] Choudhury A, Nelson LD, Teo MT, Chilka S, Bhattarai S, Johnston CF, et al. MRE11 expression is predictive of cause-specific survival following radical radiotherapy for muscle-invasive bladder cancer. Cancer Res. 2010;70(18):7017–26. 20843819 10.1158/0008-5472.CAN-10-1202 PMC2941719

[17] Harima Y, Sawada S, Miyazaki Y, Kin K, Ishihara H, Imamura M, et al. Expression of Ku80 in cervical cancer correlates with response to radiotherapy and survival. Am J Clin Oncol. 2003;26(4):e80–5. 12902903 10.1097/01.COC.0000077938.48974.59

[18] Hasegawa T, Someya M, Hori M, Matsumoto Y, Nakata K, Nojima M, et al. Expression of Ku70 predicts results of radiotherapy in prostate cancer. Strahlenther Onkol. 2017;193(1):29–37. 27465041 10.1007/s00066-016-1023-7

[19] Hu S, Qu Y, Xu X, Xu Q, Geng J, Xu J. Nuclear survivin and its relationship to DNA damage repair genes in non-small cell lung cancer investigated using tissue array. PLoS ONE. 2013;8(9):e74161. 24066112 10.1371/journal.pone.0074161 PMC3774659

[20] Saygili U, Gorkay IB, Koyuncuoglu M, Gol M, Uslu T, Erten O. The relationship between expression of Ku70 and survival in irradiated patients with endometrial carcinoma. Gynecol Oncol. 2004;95(3):518–22. 15581956 10.1016/j.ygyno.2004.08.005

[21] Soderlund Leifler K, Queseth S, Fornander T, Askmalm MS. Low expression of Ku70/80, but high expression of DNA-PKcs, predict good response to radiotherapy in early breast cancer. Int J Oncol. 2010;37(6):1547–54. 21042724 10.3892/ijo_00000808

[22] Wilson CR, Davidson SE, Margison GP, Jackson SP, Hendry JH, West CM. Expression of Ku70 correlates with survival in carcinoma of the cervix. Br J Cancer. 2000;83(12):1702–6. 11104569 10.1054/bjoc.2000.1510 PMC2363444

[23] Friesland S, Kanter-Lewensohn L, Tell R, Munck-Wikland E, Lewensohn R, Nilsson A. Expression of Ku86 confers favorable outcome of tonsillar carcinoma treated with radiotherapy. Head Neck. 2003;25(4):313–21. 12658736 10.1002/hed.10199

[24] Pavon MA, Parreno M, Leon X, Sancho FJ, Cespedes MV, Casanova I, et al. Ku70 predicts response and primary tumor recurrence after therapy in locally advanced head and neck cancer. Int J Cancer. 2008;123(5):1068–79. 18546291 10.1002/ijc.23635

[25] Lee SW, Cho KJ, Park JH, Kim SY, Nam SY, Lee BJ, et al. Expressions of Ku70 and DNA-PKcs as prognostic indicators of local control in nasopharyngeal carcinoma. Int J Radiat Oncol Biol Phys. 2005;62(5):1451–7. 16029807 10.1016/j.ijrobp.2004.12.049

[26] Joshi JS, Vora HH, Ghosh NR, Tankshali RN, Jetly DH, Trivedi TI. Nonhomologous end joining repair pathway molecules as predictive biomarkers for patients with oral squamous cell carcinoma. J Cancer Res Ther. 2021;17(4):1031–8. 34528560 10.4103/jcrt.JCRT_582_19

[27] Moeller BJ, Yordy JS, Williams MD, Giri U, Raju U, Molkentine DP, et al. DNA repair biomarker profiling of head and neck cancer: Ku80 expression predicts locoregional failure and death following radiotherapy. Clin Cancer Res. 2011;17(7):2035–43. 21349997 10.1158/1078-0432.CCR-10-2641 PMC3092475

[28] Bubendorf L, Kononen J, Koivisto P, Schraml P, Moch H, Gasser TC, et al. Survey of gene amplifications during prostate cancer progression by high-throughout fluorescence in situ hybridization on tissue microarrays. Cancer Res. 1999;59(4):803–6. 10029066

[29] Dancau AM, Simon R, Mirlacher M, Sauter G. Tissue microarrays. Methods Mol Biol. 2010;576:49–60. 19882257 10.1007/978-1-59745-545-9_4

[30] Simon R, Mirlacher M, Sauter G. Immunohistochemical analysis of tissue microarrays. Methods Mol Biol. 2010;664:113–26. 20690058 10.1007/978-1-60761-806-5_12

[31] Steurer S, Schneider J, Buscheck F, Luebke AM, Kluth M, Hube-Magg C, et al. Immunohistochemically detectable thyroglobulin expression in extrathyroidal cancer is 100% specific for thyroidal tumor origin. Ann Diagn Pathol. 2021;54:151793. 34425503 10.1016/j.anndiagpath.2021.151793

[32] Huber W, Carey VJ, Gentleman R, Anders S, Carlson M, Carvalho BS, et al. Orchestrating high-throughput genomic analysis with Bioconductor. Nat Methods. 2015;12(2):115–21. 25633503 10.1038/nmeth.3252 PMC4509590

[33] Kassambara A, Kosinski M, survminer. Drawing Survival Curves using ‘ggplot2’. R package version 0.4.3. https://CRANR-projectorg/package=survminer. 2018.

[34] Therneau TM, Grambsch PM. Modeling Survival Data: Extending the Cox Model. Springer, New York ISBN 0-387-98784-3. 2000.

[35] Wei T, Simko V. R package corrplot: visualization of a correlation matrix (Version. 84). https://githubcom/taiyun/corrplot. 2017.

[36] Wickham H. Reshaping data with the reshape Package. J Stat Softw. 2007;21(12):1–20. http://www.jstatsoft.org/v21/i12/. .

[37] Castellsague X, Alemany L, Quer M, Halec G, Quiros B, Tous S, et al. HPV involvement in Head and Neck cancers: Comprehensive Assessment of biomarkers in 3680 patients. J Natl Cancer Inst. 2016;108(6):djv403. 26823521 10.1093/jnci/djv403

[38] Nauta IH, Heideman DAM, van der Brink A, Bloemena E, Koljenovic S, et al. The unveiled reality of human papillomavirus as risk factor for oral cavity squamous cell carcinoma. Int J Cancer. 2021;149(2):420–30. 33634865 10.1002/ijc.33514 PMC8251537

[39] Simoens C, Gorbaslieva I, Gheit T, Holzinger D, Lucas E, Ridder R, et al. HPV DNA genotyping, HPV E6*I mRNA detection, and p16(INK4a)/Ki-67 staining in Belgian head and neck cancer patient specimens, collected within the HPV-AHEAD study. Cancer Epidemiol. 2021;72:101925. 33839457 10.1016/j.canep.2021.101925

[40] Taberna M, Resteghini C, Swanson B, Pickard RK, Jiang B, Xiao W, et al. Low etiologic fraction for human papillomavirus in larynx squamous cell carcinoma. Oral Oncol. 2016;61:55–61. 27688105 10.1016/j.oraloncology.2016.08.009

[41] Tagliabue M, Mena M, Maffini F, Gheit T, Quiros Blasco B, Holzinger D et al. Role of human papillomavirus infection in Head and Neck Cancer in Italy: the HPV-AHEAD study. Cancers (Basel). 2020;12(12). 10.3390/cancers12123567 PMC7760748 33260360

[42] Wittekindt C, Wuerdemann N, Gattenlohner S, Brobeil A, Wierzbicka M, Wagner S, et al. The role of high-risk human papillomavirus infections in laryngeal squamous cell carcinoma. Eur Arch Otorhinolaryngol. 2017;274(11):3837–42. 28861601 10.1007/s00405-017-4718-1

[43] Kocher S, Zech HB, Krug L, Gatzemeier F, Christiansen S, Meyer F, et al. A lack of effectiveness in the ATM-Orchestrated DNA damage response contributes to the DNA repair defect of HPV-Positive Head and Neck Cancer cells. Front Oncol. 2022;12:765968. 35719921 10.3389/fonc.2022.765968 PMC9204973

[44] Liu Q, Ma L, Jones T, Palomero L, Pujana MA, Martinez-Ruiz H, et al. Subjugation of TGFbeta Signaling by Human Papilloma Virus in Head and Neck squamous cell carcinoma shifts DNA repair from homologous recombination to alternative end joining. Clin Cancer Res. 2018;24(23):6001–14. 30087144 10.1158/1078-0432.CCR-18-1346

[45] Ma J, Setton J, Morris L, Albornoz PB, Barker C, Lok BH, et al. Genomic analysis of exceptional responders to radiotherapy reveals somatic mutations in ATM. Oncotarget. 2017;8(6):10312–23. 28055970 10.18632/oncotarget.14400 PMC5354661

[46] Mari PO, Florea BI, Persengiev SP, Verkaik NS, Bruggenwirth HT, Modesti M, et al. Dynamic assembly of end-joining complexes requires interaction between Ku70/80 and XRCC4. Proc Natl Acad Sci U S A. 2006;103(49):18597–602. 17124166 10.1073/pnas.0609061103 PMC1693708

[47] Shao Z, Davis AJ, Fattah KR, So S, Sun J, Lee KJ, et al. Persistently bound Ku at DNA ends attenuates DNA end resection and homologous recombination. DNA Repair (Amst). 2012;11(3):310–6. 22265216 10.1016/j.dnarep.2011.12.007 PMC3297478

[48] Soutoglou E, Dorn JF, Sengupta K, Jasin M, Nussenzweig A, Ried T, et al. Positional stability of single double-strand breaks in mammalian cells. Nat Cell Biol. 2007;9(6):675–82. 17486118 10.1038/ncb1591 PMC2442898

[49] Mansour WY, Schumacher S, Rosskopf R, Rhein T, Schmidt-Petersen F, Gatzemeier F, et al. Hierarchy of nonhomologous end-joining, single-strand annealing and gene conversion at site-directed DNA double-strand breaks. Nucleic Acids Res. 2008;36(12):4088–98. 18539610 10.1093/nar/gkn347 PMC2475611

[50] Wang M, Wu W, Wu W, Rosidi B, Zhang L, Wang H, et al. PARP-1 and Ku compete for repair of DNA double strand breaks by distinct NHEJ pathways. Nucleic Acids Res. 2006;34(21):6170–82. 17088286 10.1093/nar/gkl840 PMC1693894

[51] Chen Y, Zhao Y, Yang X, Ren X, Huang S, Gong S, et al. USP44 regulates irradiation-induced DNA double-strand break repair and suppresses tumorigenesis in nasopharyngeal carcinoma. Nat Commun. 2022;13(1):501. 35079021 10.1038/s41467-022-28158-2 PMC8789930

[52] Chen X, Chen C, Luo C, Liu J, Lin Z. Discovery of UMI-77 as a novel Ku70/80 inhibitor sensitizing cancer cells to DNA damaging agents in vitro and in vivo. Eur J Pharmacol. 2024;975:176647. 38754534 10.1016/j.ejphar.2024.176647

[53] Prigge ES, Arbyn M, von Knebel Doeberitz M, Reuschenbach M. Diagnostic accuracy of p16(INK4a) immunohistochemistry in oropharyngeal squamous cell carcinomas: a systematic review and meta-analysis. Int J Cancer. 2017;140(5):1186–98. 27859245 10.1002/ijc.30516

