# Supplementary material for: Tissue microarray analyses of the essential DNA repair factors ATM, DNA-PKcs and Ku80 in head and neck squamous cell carcinoma
Source: Radiat Oncol. 2024 Oct 30;19:150. doi: 10.1186/s13014-024-02541-3 (PMC11523811; doi:10.1186/s13014-024-02541-3)
Supplement: Supplementary file 1 — Supplementary Material 1 [file 13014_2024_2541_MOESM1_ESM.pdf]

Supplementary Figures

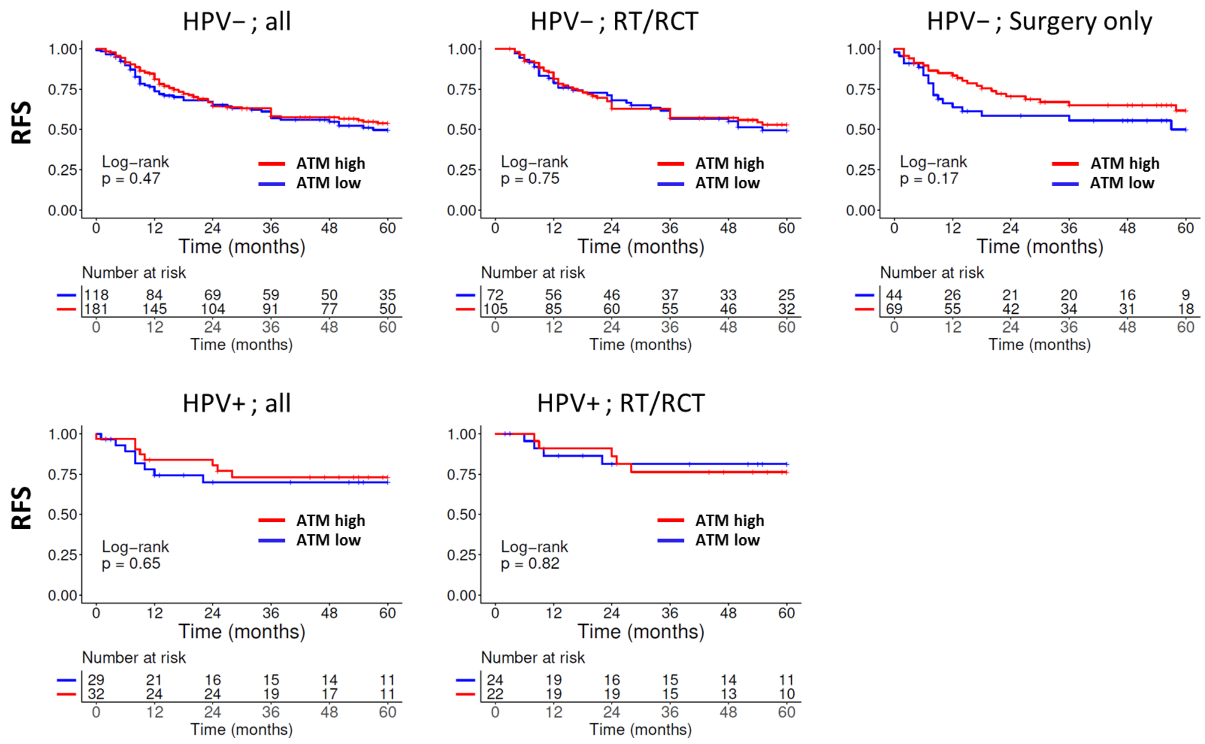

Supplementary Figure 1. Recurrence free survival in dependence of ATM expression and treatment. ATM-expression was categorized by staining scores as *low* (*absent & weak*) or *high* (*moderate & strong*). The low numbers of p16-positive OPSCC treated solely by surgery prevented a meaningful analysis.

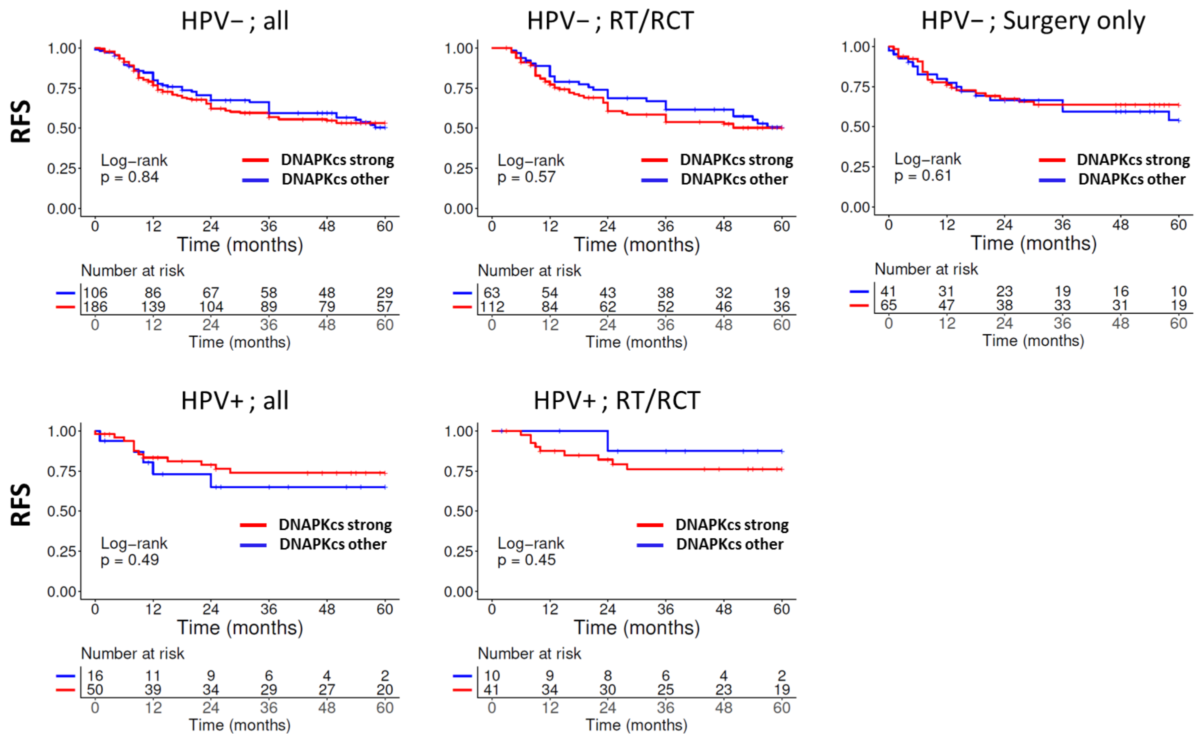

Supplementary Figure 2. Recurrence free survival in dependence of DNA-PKcs expression and treatment. Expression was categorized by staining scores as *strong* or *other*. The low numbers of p16-positive OPSCC treated solely by surgery prevented a meaningful analysis.

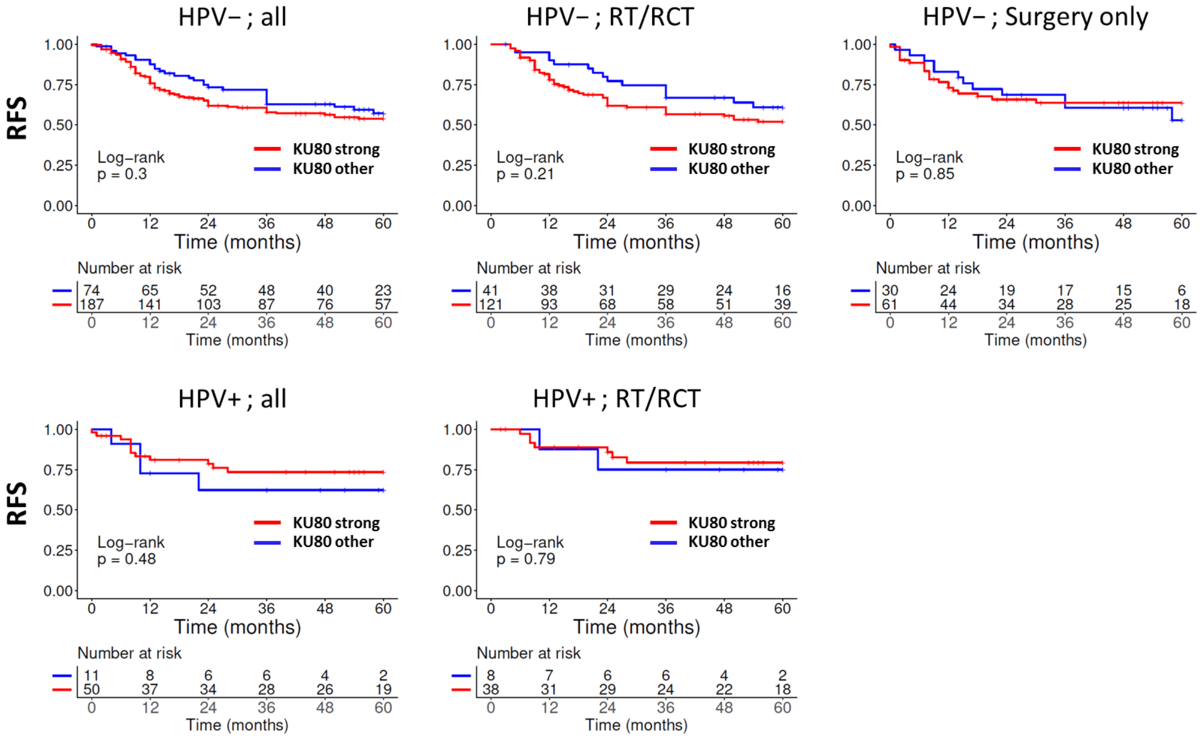

Supplementary Figure 3. Recurrence free survival in dependence of Ku80 expression and treatment. Expression was categorized by staining scores as *strong* or *other*. The low numbers of p16-positive OPSCC treated solely by surgery prevented a meaningful analysis.

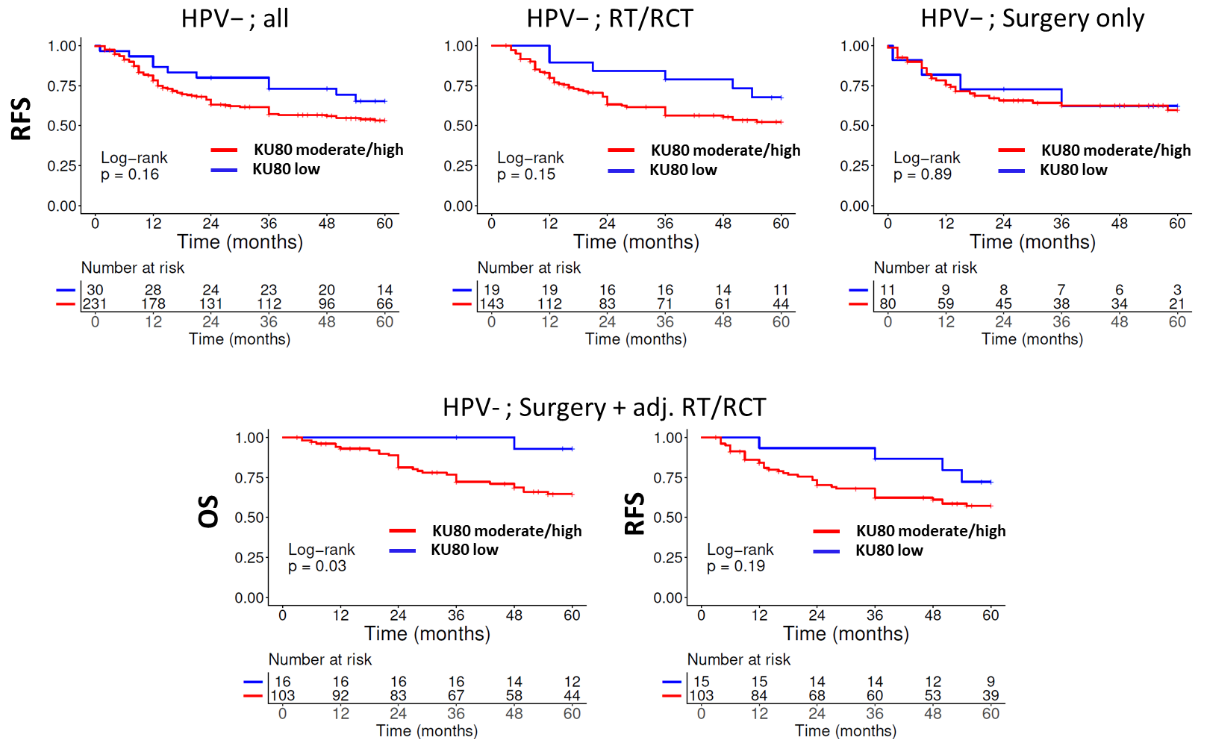

Supplementary Figure 4. Survival in dependence of Ku80 staining intensity and treatment. Expression was categorized by staining intensities as either *low* (0,1) or *high* (2,3) with a threshold of >30 % of tumor cells with at least intermediate staining intensity necessary to be assorted to the higher category. Top: Recurrence free survival after treatment as indicated. Bottom: Overall and Recurrence free survival of patients with HPV-negative tumors treated by surgery and adjuvant RT/RCT.
